# Oestradiol-17β plasma concentrations after intramuscular injection of oestradiol benzoate or oestradiol cypionate in llamas (*Lama glama*)

**DOI:** 10.1186/1751-0147-52-13

**Published:** 2010-02-11

**Authors:** María V Cavilla, Carolina P Bianchi, Marcelo A Aba

**Affiliations:** 1 Área de Endocrinología, Facultad de Ciencias Veterinarias, UNCPBA, Campus Universitario, Paraje Arroyo Seco s/n, Tandil-7000, Buenos Aires, Argentina

## Abstract

**Background:**

Llamas (*Lama glama*) are induced ovulators and the process of ovulation depends on dominant follicular size. In addition, a close relationship between behavioural estrus and ovulation is not registered in llamas. Therefore, the exogenous control of follicular development with hormones aims to predict the optimal time to mate. Oestradiol-17β (E_2_) and its esters are currently used in domestic species, including camelids, in synchronization treatments. But, in llamas, there is no reports regarding the appropriate dosages to be used and most protocols have been designed by extrapolation from those recommended for other ruminants. The aim of the present study was to characterize plasma E_2 _concentrations in intact female llamas following a single intramuscular (i.m.) injection of two oestradiol esters: oestradiol benzoate (EB) and oestradiol cypionate (ECP).

**Methods:**

Twelve non pregnant and non lactating sexually mature llamas were i.m. injected on day 0 with 2.5 mg of EB (EB group, n = 6) or ECP (ECP group, n = 6). Blood samples were collected immediately before injection, at 1, 6, 12, 24 h after treatment and then daily until day 14 post injection. Changes in hormone concentrations with time were analyzed in each group by analysis of variance (ANOVA) using a repeated measures (within-SS) design. Plasma E_2 _concentrations and area under the concentration-time curve (AUC) values were compared between groups by ANOVA. In all cases a Least-Significant Difference test (LSD) was used to determine differences between means. Hormonal and AUC data are expressed as mean ± S.E.M.

**Results:**

Peak plasma E_2 _concentrations were achieved earlier and were higher in EB group than in ECP group. Thereafter, E_2 _returned to physiological concentrations earlier in EB group (day 5) than in ECP group (day 9). Although plasma E_2 _profiles differed over time among groups there were no differences between them on AUC values.

**Conclusions:**

The i.m. injection of a single dose of both oestradiol esters resulted in plasma E_2 _concentrations exceeding physiological values for a variable period. Moreover, the plasma E_2 _profiles observed depended on the derivative of oestradiol administered. This basic information becomes relevant at defining treatment protocols including oestrogens in llamas.

## Background

Llamas (*Lama glama*) are ruminants with physiological peculiarities, particularly regarding their reproductive physiology. Ovarian follicles develop in a wave-like pattern as in other ruminants: cows [1]; ewes [2]; goats [3], but the process of ovulation requires the stimulus of mating and ejaculation [4-8]. Moreover, only growing, dominant follicles ≥ 7 mm in diameter present at the time of mating have the ability to ovulate [9]. In the absence of ovulation, successive waves of follicular growth and regression that generally overlap, proceed [10,11]. The oestrogens produced during those waves determine long periods of receptivity (≥ 30 days), interrupted by short stages of male rejection [4,12]. Thus, a set of signals indicative of the presence of a mature follicle is not registered in the llama [5,6,11]. Furthermore, the female might accept the male in the absence of a qualified follicle resulting in ovulation failure. Therefore, the control of follicular development with exogenous hormones assuring the presence of a healthy mature follicle at a fixed time after treatment would enable the implementation of reproductive technologies leading to an increase in productivity.

Oestrogens are a family of steroid hormones involved in female reproductive processes regulating and sustaining sexual development and reproductive function. Oestradiol-17β (E_2_) is the most abundant and active of the endogenous oestrogens produced by the ovary [13]. In addition, oestradiol esters are a group of synthetic oestrogens that includes: oestradiol benzoate (EB), oestradiol valerate (EV) and oestradiol cypionate (ECP). Oestradiol-17β and EB are commonly used to induce follicular regression and to synchronize wave emergence and ovulation in progestin-treated cattle [14-17]. The inclusion of ECP in hormonal regimens has increased in recent years as in some countries is the only ester available and licensed for use in cattle, but its success to hasten wave emergence has been relatively variable to date [18,19]. The time to wave emergence after E_2_treatment in cattle, appears to depend on the dose and formulation administered. It appears that the greater the circulating concentrations of E_2, _or the time to return to physiological range, the greater are follicular and FSH suppression [16,20-22]. Thus the use of an "optimal" dose or formulation of E_2 _in hormonal regimens is emphasized by the previous observations. Conversely, in camelids information regarding the effects of oestrogens on follicular activity is scanty and controversial [23-26]. In addition, there is no reports regarding the appropriate dosages to be used in llamas and most protocols have been designed by extrapolation from those recommended for other ruminants. While establishing those dose regimens, it has never been considered that camelids show pharmacological differences for the metabolism of several drugs as compared to other ruminants [27-30].

The study hereby reported, was designed to provide a comparative characterization of plasma E_2 _concentrations following a single i.m. injection of two oestradiol esters (EB and ECP). This information would provide basic knowledge useful for a more rational use of these drugs in the species.

## Methods

### Animals and treatments

The experimental design and animal care were performed in compliance with regulations set by the Animal Welfare Committee at the Faculty of Veterinary Sciences, UNCPBA, Tandil, Argentina (37°S, 60°W) where activities were carried out. Animals were kept isolated from males and fed pasture hay and had free access to water during the entire experimental period. Twelve sexually mature, non pregnant and non lactating female llamas (107.5 ± 17.68 Kg) were randomly divided into two treatment groups: EB (n = 6) and ECP (n = 6). On day 0 all animals received a single i.m. injection (0 h) of 2.5 mg of oestradiol benzoate (EB, Benzoato de Estradiol Syntex^®^, Syntex SA, Buenos Aires, Argentina) or oestradiol cypionate (ECP, ecp estradiol^®^, König, Buenos Aires, Argentina) into the hind leg in the region of the semitendinosus muscle. The dose of EB was selected based on previous studies in llamas and alpacas [24,31]. In general, a larger dose of EB has been used in camelids as compared to that used in synchronization programs in cattle [15,24,26,31], probably in relation to a higher metabolic rate reported in camelids for different drugs [27-30]. In addition, to allow a direct comparison between esters and due to the lack of reports using ECP in the specie the same dose was used for ECP.

### Blood sampling

Blood samples were collected into heparinized tubes immediately before injection, at 1, 6, 12, 24 h after treatment, and then daily until day 14 post injection. In order to minimize damage to the jugular veins due to the high frequency sampling protocol, venipuncture was performed alternately at high, medium and low positions on the left and right sides of the neck as previously described by Aba *et al*. [32]. Plasma samples were separated by centrifugation and stored at -20°C until analyzed.

### Hormone determinations

Oestradiol-17β plasma concentrations were measured using an RIA Kit (Siemens Medical Solutions Diagnostics, Los Angeles, CA, USA) reported for use with bovine plasma [33], and validated for use with llama plasma after minor modifications [6]. Plasma and standards were previously extracted using diethyl ether (Merck, Buenos Aires, Argentina). The intra-assay coefficient of variation was below 10% for concentrations between 5.6 and 180 pmol l^-1^. The inter-assay coefficient of variation, calculated from the precision profiles of five standard curves, was below 6% for concentrations between 5.6 and 180 pmol l^-1^. The lowest amount of E_2 _detectable was 5.6 pmol l^-1^. Plasma progesterone (P_4_) concentrations were measured using an RIA Kit (Siemens Medical Solutions Diagnostics, Los Angeles, CA, USA) previously validated for use with llama plasma [34]. The sensitivity of the assay was 0.3 nmol l^-1 ^and the intra-assay coefficient of variation was below 13% for concentrations between 0.4 and 128 nmol l^-1^. All samples were measured in duplicates. Hormone concentrations are expressed in SI units. To convert from pmol l^-1 ^to pg ml^-1 ^and from nmol l^-1 ^to ng ml^-1^, a factor of 3.7 for oestradiol-17β and 3.2 for progesterone should be used.

### Analysis of data

In order to establish a physiological plasma E_2 _concentration upper limit before treatment, twice the standard deviation (SD) was added to the general mean as previously described by Vynckier *et al*. [35]. Any concentration above (P < 0.05) this limit was considered pharmacological. Changes in hormone concentrations with time on each treatment group were analyzed by analysis of variance (ANOVA) using a repeated measures (within-SS) design. Mean E_2 _concentrations at each time were statistically compared among groups by ANOVA. In all cases, a Fisher's Least-Significant Difference test (LSD) was used to determine differences between means. Log-transformation was used for plasma E_2 _concentrations before analyses to stabilize variances. The area under the concentration-time curve (AUC) from time zero to the last measurable concentration was calculated by the trapezoidal rule [36]. The AUC values obtained after the injection of both esters was statistically compared between groups by ANOVA. Statistical analyses were carried out using the Statistica/W, release 4.0 software package (Statsoft Inc., Tulsa, OK, USA, 1993). Statistical significance was set at P < 0.05. Hormonal and AUC data are expressed as mean ± S.E.M unless otherwise specified.

## Results

### Physiological plasma E_2 _concentrations

Oestradiol-17β plasma concentrations varied widely between animals at the beginning of the study (ranging from 13.7 to 44.7 pmol l^-1^). Mean plasma E_2 _concentration before injection (considering all females) was 33.1 ± 11.7 pmol l^-1 ^(mean ± SD). The physiological upper limit for plasma E_2 _concentrations was established at 56.5 pmol l^1^.

### Plasma E_2 _concentrations and AUC values after oestradiol esters injection

**Figure 1:**
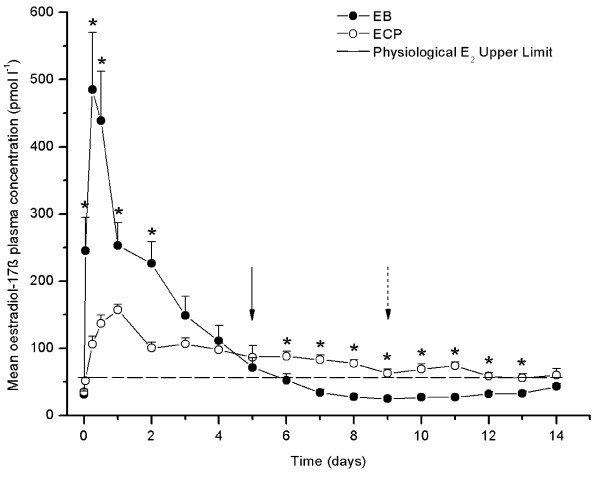
**Mean plasma oestradiol 17-β concentration in llamas injected intramuscularly with oestradiol benzoate or oestradiol cypionate**. Mean plasma oestradiol 17-β (E_2_) concentration immediately before injection (0 h), at 1, 6, 12, 24 h post injection, and then daily until day 14 after a single intramuscular injection of 2.5 mg of oestradiol benzoate (EB group; closed circle) or oestradiol cypionate (ECP group; open circle) in llamas (n = 6/group). The broken line indicates the physiological plasma E_2 _concentrations upper limit established. ^(*)^Mean values differ between groups at P < 0.05. Within treatments groups, a solid (EB group) or dashed (ECP group) arrow indicates the day on which plasma E_2 _concentrations returned to physiological values (P > 0.05).

The administration of both formulations resulted in plasma E_2_concentrations largely exceeding physiological values. Mean plasma E_2_concentrations during 14 days after i.m. injection of EB and ECP are shown in Figure 1. In EB group, E_2 _plasma concentrations increased sharply (P < 0.0001) by 1 h after injection, reaching peak concentrations (484.9 ± 85.6 pmol l^-1^) by 6 h after injection. Oestradiol-17β plasma concentrations remained elevated until 12 h and then followed a biphasic decline. Firstly, E_2 _concentrations underwent a sharp decrease between 12 h and day 1, remained relatively stable between days 1 and 2 and steadily decreased onwards returning to physiological values by day 5 post injection. In ECP group, E_2 _concentrations increased slowly from 6 h after injection (P < 0.0001) until peak concentrations (157.1 ± 8.7 pmol l^-1^) were reached on day 1. Thereafter, plasma E_2 _concentrations declined slowly, until concentrations no longer distinguishable from physiological were attained on day 9. Subsequently, plasma E_2 _concentrations fluctuated close to physiological values until the end of the study.

Although plasma E_2 _concentrations profiles differ according to treatment, the AUC values obtained following injection were similar between groups (EB: 1336.01 ± 157.4 pmol*day/l; ECP: 1189.78 ± 87.44 pmol*day/l; P = 0.5306). Thus, mean E_2 _concentrations in EB group were higher than in ECP group at all times beyond the 0 h sample until day 2 with a rapid fell onwards. In contrast, in ECP group mean E_2 _concentrations experienced a slower increase, but more prolonged in time, attaining values higher than in EB group between day 6 and day 13 inclusive. On days 3 to 5 and on day 14 plasma E_2 _concentrations were similar among groups. Plasma P_4 _concentrations remained bellow 3 nmol l^-1 ^in all females during the entire period of study.

## Discussion

Plasma E_2 _concentrations and its variability between animals recorded before treatment in the present study (range from 13.7 to 44.7 pmol l^-1^) are consistent with previous reports in llamas [6,37,38]. This observation indicates animals were at different stages of follicular development at the time of treatment, as it has been established a close relationship between follicular activity and E_2 _secretion in llamas [5,6,38,39]. In addition, the plasma E_2 _concentration established as the physiological upper limit in the present study was somehow in agreement to that previously registered during the follicular phase (17 to 47 pmol l^-1^) in llamas [38]. The fact that plasma P_4 _concentrations remained bellow 3 nmol l^-1 ^in all cases indicates that no ovulations occurred during the study [6].

To our knowledge, this is the first report showing the plasma E_2 _profile following a single i.m. injection of ECP and its comparison to the profile obtained after injection of EB in llamas. Treatment with both oestradiol derivatives induced plasma E_2 _concentrations exceeding those considered physiological for the species. Moreover, the plasma E_2_profiles recorded in the present study clearly depended on the ester administered and were consistent with that registered in cows by Vynckier *et al*. [35]. Plasma E_2 _concentrations increased rapidly and peaked earlier in EB group with values three-times higher than in ECP group. Similar results were previously observed after EB injection in bovines [21,22].

Peak plasma E_2 _concentrations achieved after EB injection in the present study were higher than those recorded before in camelids [24,31] and were attained earlier than observed by others [24] in alpacas. Although there is no clear explanation for the divergences registered between studies, it could be speculated that differences in blood sampling schedule, sensitivity of hormone assays and formulation of hormonal products, may be responsible for the discrepancy.

Once peak plasma E_2 _concentrations were reached, the declination rate also differed between esters. The biphasic decline of plasma E_2_concentrations until physiological values on day 5 in EB group is in agreement with previous reports in alpacas and cows [24,35]. As stated by Vynckier *et al*. [35] these biphasic decline suggest an initial phase of distribution followed by an elimination phase. In contrast, in ECP group, the decreasing phase of the curve was shorter and plasma E_2 _concentrations fluctuated close to physiological values from day 9 onwards. Although the pattern of decline observed for ECP in our study is consistent with that recorded in cows by Vynckier *et al*. [35], they showed that concentrations returned to physiological values slightly earlier (day 7) in this species. The slower return to physiological values in ECP compared to EB group indicates a longer half-life for ECP as has been registered previously in cattle [17,19,35].

From the analysis of plasma E_2 _concentrations, it is apparent that ECP was absorbed at a lower rate than EB, but persisted elevated in plasma for a longer period (8 and 4 days, respectively). Besides the different rates of absorption, the same amount of drug was released from the injection site for both esters, since by the end of the study the AUCs values were similar between groups. These findings are consistent with the fact that the esterification process prolongs the half-life of E_2_. Apparently, the ester injected influences the mode that E_2 _is released from the injection site. Thus, there is an inverse relationship between the extension of the ester chain and its water solubility and consequently, on its absorption rate. Once in circulation, the esters are hydrolyzed to E_2_, the active hormone. Consequently, the duration of action appears to depend on the rate of absorption more than the metabolism [40].

It has been suggested in llamas that hormonal treatments with progestogens are effective in completely prevent follicular development for a period of up to 7 days with the lowest follicular activity registered between days 5 and 7 after beginning of treatment [32-38]. Thereafter, most attempts to control follicular activity includes short protocols (5 to 7 days) and a combination of a progestogen and oestrogens. According to the results hereby presented, E_2 _plasma concentrations would remain at pharmacological concentrations beyond the day of progestogen withdrawal after ECP treatment. Conversely, plasma E_2 _concentrations would have return to physiological values before the end of the synchronization protocol after EB injection. Whether this might influence the outcome of the synchronization treatment or not remains to be elucidated.

## Conclusions

To our knowledge, this is the first study that provides a characterization of the plasma E_2 _concentrations profile following a single i.m. injection of ECP and a direct comparison to that obtained after EB injection in intact female llamas. Both esters effectively induced pharmacological E_2 _concentrations, however, plasma E_2 _profiles depended on the formulation administered. Peak plasma E_2 _concentrations were higher and were attained earlier after EB injection compared to ECP with an earlier return to physiological values. This basic information becomes relevant at defining treatment protocols including oestrogens in llamas.

## Competing interests

The authors declare that they have no competing interests.

## Authors' contributions

MVC participated in developing the design of the experiment, carried out the experiment, gathered and analyzed the data, and drafted the manuscript. CPB contributed in carrying out the experiment and with contents of the manuscript. MAA as the director of the doctoral project of MVC designed the experiment, contributed in carrying out the experiment, with the analysis of the data and revising critically the contents of the manuscript. All authors have read and approved the final manuscript.
